# Impact of coronary artery revascularization on long-term outcome in hypertrophic cardiomyopathy patients: a nationwide population-based cohort study

**DOI:** 10.1038/s41598-023-33344-3

**Published:** 2023-04-19

**Authors:** Tae-Min Rhee, Hyung-Kwan Kim, Bong-Seong Kim, Kyung-Do Han, Hyun-Jung Lee, In-Chang Hwang, Heesun Lee, Jun-Bean Park, Yeonyee E. Yoon, Yong-Jin Kim, Goo-Yeong Cho

**Affiliations:** 1 Division of Cardiology, Department of Internal Medicine, Seoul National University College of Medicine, Cardiovascular Center, Seoul National University Hospital, 101 Daehak-Ro, Jongno-Gu, Seoul, 03080 Korea; 2 Department of Statistics and Actuarial Science, The Soongsil University, Seoul, Republic of Korea; 3 Cardiovascular Center and Department of Internal Medicine, Seoul National University Bundang Hospital, Seongnam, Gyeonggi Republic of Korea; 4 Division of Cardiology, Department of Internal Medicine, Seoul National University Hospital Healthcare System Gangnam Center, Seoul, Republic of Korea

**Keywords:** Cardiology, Cardiovascular biology

## Abstract

Limited data are available on the long-term outcomes in patients with hypertrophic cardiomyopathy (HCM) patients with significant coronary artery disease (CAD) requiring revascularization. We investigated the risk of cardiovascular outcomes in HCM patients who underwent coronary revascularization compared to the control group without HCM. HCM patients aged ≥ 20 years were enrolled from the Korean National Health Insurance Database. Information on the diagnosis and previous medical history was obtained from the claims data. Cardiovascular outcomes were identified during 8-year after coronary revascularization in HCM patients (HCM group) and matched controls without HCM (non-HCM control group). A total of 431 patients in the HCM group and 1968 in the non-HCM control group were analyzed. The risk of all-cause death, cardiovascular death, sudden cardiac death (SCD), ischemic stroke, and hospitalization due to heart failure was significantly higher in the HCM group than in the non-HCM group, with prominent risk increase of cardiovascular death (adjusted hazard ratio [HR] 2.27, 95% confidence interval [CI] 1.63–3.15, *P* < 0.001) and ischemic stroke (adjusted HR 2.38, 95% CI 1.55–3.64, *P* < 0.001). Beyond 1-year after revascularization, the HCM group still had a significantly higher risk of cardiovascular death, SCD, and ventricular fibrillation/tachycardia compared to the non-HCM group. Mortality and major cardiovascular outcomes occurred more frequently in HCM patients with significant CAD requiring revascularization, compared to the matched non-HCM control group. Active and regular surveillance for concomitant risk factors and relevant intervention are warranted in HCM patients at increased risk for CAD.

## Introduction

Hypertrophic cardiomyopathy (HCM) is one of the most common inherited cardiomyopathies, with an estimated global prevalence of 0.2–0.5%^1^. Thanks to recent advances in the management strategy, the majority of patients with HCM live their own lives, largely free from major complications including atrial fibrillation, stroke, heart failure (HF), and sudden cardiac death (SCD)^2,3^, resulting in an annual mortality rate of < 1%^4^. However, myocardial ischemia has emerged as an important comorbidity in HCM patients^5^ and frequently originates at the microvascular level with structural abnormalities in the intramural coronary arterioles^1,6^. Moreover, HCM patients in the middle or old age are susceptible to epicardial atherosclerotic coronary artery disease (CAD). One-third of patients with HCM who underwent coronary computed tomography angiography (CCTA) for chest pain had clinically significant epicardial CAD as well as plaques with unfavorable characteristics^7,8^. Although the presence of severe epicardial CAD is a well-known risk factor for mortality and SCD in HCM patients^9^, it has not been adequately addressed and the effect of coronary revascularization, i.e., coronary artery bypass graft (CABG) surgery or percutaneous coronary intervention (PCI), on the prognosis of HCM has not been well-explored in a sizable number of patients. Using the Korean nationwide population-based cohort, we sought to investigate the long-term risk of major cardiovascular outcomes in patients with HCM (HCM group) who underwent coronary revascularization compared to a control group without HCM (non-HCM control group).

## Methods

### Data source and study population

This nationwide population-based cohort study analyzed the data extracted from the Korean National Health Insurance Service (NHIS) database. The structure and characteristics of the data source have been described previously in detail^10^. The NHIS is a mandatory universal health insurance program that contains medical information including demographics, socioeconomic status, medical treatments and procedures performed, and diagnoses of diseases according to the 10th revision of the International Classification of Diseases (ICD-10), of the entire Korean population^11–20^.

The study protocol conformed to the ethical guidelines of the Declaration of Helsinki. Since all the patients' records and information were completely anonymized and de-identified before cohort establishment, this study was exempt from informed consent and waived off from the approval after careful review by the Institutional Review Board of Seoul National University Hospital (E-2012-111-1183).

### Diagnosis of hypertrophic cardiomyopathy

HCM was defined as (1) at least one hospitalization or outpatient clinic visit with the ICD-10 codes I42.1 or I42.2, and (2) registration in the Rare Intractable Disease (RID) program (RID code V127). Since 2006, the NHIS operates the RID program to provide special medical aid benefits to patients with diseases covered in this program. The government-implemented RID program is a welfare policy extending health insurance coverage to 90% of all medical expenses claimed by these patients. Thus, the RID program is strictly monitored and verified in accordance with an act established by the Ministry of Health and Welfare by investigating clinical and imaging evidence and physician’s certification, and by independent reviews by medical experts and health insurance professionals^10,12^. The diagnostic accuracy of HCM using the ICM-10 and RID codes was previously validated using our institutional database, showing an accuracy of 92.6%^12^.

### Construction of study cohort

**Figure 1:**
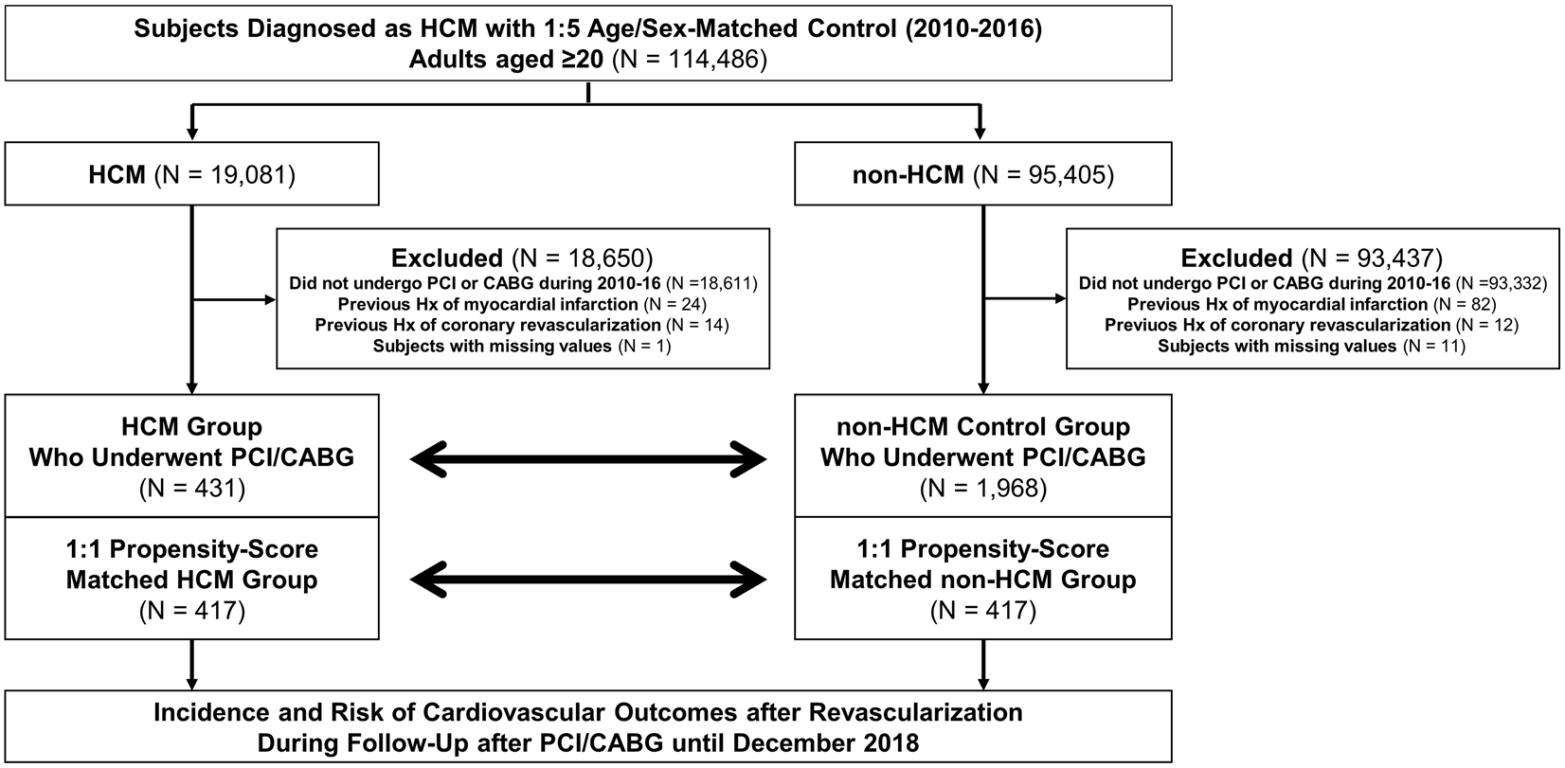
Study flow. From the Korean National Health Information Database, participants aged ≥ 20 were screened and patients with HCM were identified during 2010–2016. Control group without HCM was selected by 1:5 matching of age and sex. The HCM group and non-HCM control group who underwent PCI or CABG during the study period were analyzed. Abbreviations: CABG, coronary artery bypass graft; HCM, hypertrophic cardiomyopathy; Hx, history; PCI, percutaneous coronary intervention.

From the NHIS database, we screened participants aged ≥ 20 between January 1, 2010 and December 31, 2016 (Fig. 1) and identified patients with HCM using dedicated ICD-10 codes and registration in the RID program as described above. By 1:5 age/sex-matching, the non-HCM control group without a previous history of HCM was selected from the same NHIS database. We then excluded participants who 1) did not undergo PCI or CABG from 2010–2016, 2) had a previous history of MI or CABG or/and PCI, and 3) had missing values in their records. To adjust for the imbalance of baseline characteristics between the HCM group and the non-HCM control group, each HCM patient was matched to one non-HCM control participant with a caliper for nearest-neighbor matching within 0.2 standard deviations^21^. The participants were followed up for a median of 4.25 years (Q1–Q3, 2.61–5.88 years) in the HCM group and 4.84 years (Q1–Q3, 3.11–6.74 years) in the non-HCM control group until the end of December 2018.

### Definition of covariates and study outcomes

The detailed definitions of the covariates and study outcomes, which were validated in the previous publications, are described in the Table S1^10–15^. Data on age and sex were acquired from the resident identification numbers. Income level was dichotomized at the lowest 20% and presented as a categorical variable. The outcomes of interest were individual cardiovascular outcomes including all-cause death, cardiovascular death, SCD, ischemic stroke, hospitalization due to HF, ventricular fibrillation/tachycardia, MI, and repeat revascularization during the follow-up.

### Statistical analysis

Categorical variables were presented as numbers and relative frequencies (percentages), and continuous variables as mean ± standard deviation. The chi-square test and the independent sample t-test were used for comparison. Incidence rates (IRs) of clinical outcomes were calculated as the number of events per 1000 person-years. The cumulative incidence of each clinical outcome was compared between the HCM and the non-HCM control groups using Kaplan–Meier censoring estimates and the log-rank test. Hazard ratios (HRs) with 95% confidence intervals (CIs) were calculated using univariable and multivariable Cox proportional hazard models. The adjusted HRs were calculated using a multivariable model with age, sex, low income, revascularization due to acute MI, revascularization after CCTA, hypertension, diabetes mellitus (DM), dyslipidemia, non-valvular atrial fibrillation (AF), history of previous stroke, and history of congestive HF as covariates.

The results obtained were validated in the propensity score-matched (PSM) cohort. Variables that could potentially affect the clinical outcomes (i.e., age, sex, low income, revascularization due to acute MI, revascularization after CCTA, hypertension, DM, dyslipidemia, non-valvular AF, history of previous stroke, and history of congestive HF) were included in the logistic regression model to produce propensity scores for each participant. A 1:1 matching without replacement was performed by the nearest-neighbor matching algorithm with a caliper of 0.2 standard deviation, yielding a PSM cohort consisting of 417 HCM patients matched with 417 non-HCM control patients. Subgroup analyses according to age, sex, DM, revascularization due to acute MI, and non-valvular AF were performed. *P* values were two-sided, and a *P* value of < 0.05 was considered statistically significant. Statistical analyses were performed using SAS version 9.4 (SAS Institute, Cary, NC, USA) and Stata statistical software version 14 (StataCorp, College Station, TX, USA).

## Results

### Baseline characteristics of the study population

The baseline profiles of the study population before and after PSM are described in Table 1. Before PSM, the HCM group (n = 431) was younger and had a higher proportion of non-valvular AF, history of previous stroke, and congestive HF than the non-HCM control group (n = 1968). The number of patients who underwent coronary revascularization due to acute MI was higher in the non-HCM control than in the HCM groups, while the number of patients who had CCTA results before revascularization was significantly higher in the HCM group. After PSM, the differences between all the matched variables were well-balanced with absolute standardized difference < 0.1. In the HCM group, the median duration between the first diagnosis of HCM and coronary revascularization was 1142 days (Q1–Q3, 339–2224 days).

**Table 1:** Baseline characteristics of before and after propensity-score matched population.

	Before PS-matching				After PS-matching			
	HCM Group (n = 431)	non-HCM Control group (n = 1968)	*P*	ASD	HCM Group (n = 417)	non-HCM Control group (n = 417)	*P*	ASD
Age, years	64.9 ± 10.3	67.8 ± 9.6	< 0.001	0.287	65.3 ± 10.1	65.7 ± 10.0	0.561	0.040
Male, n (%)	297 (68.9)	1394 (70.8)	0.428	0.042	289 (69.3)	287 (68.8)	0.881	0.010
Low income status (bottom 20%), n (%)	69 (16.0)	434 (22.1)	0.005	0.154	67 (16.1)	65 (15.6)	0.850	0.013
Comorbidities and risk factors								
Diabetes mellitus, n (%)	143 (33.2)	721 (36.6)	0.176	0.073	140 (33.6)	126 (30.2)	0.298	0.072
Hypertension, n (%)	327 (75.9)	1456 (74.0)	0.417	0.044	316 (75.8)	333 (79.9)	0.157	0.098
Dyslipidemia, n (%)	372 (86.3)	1629 (82.8)	0.074	0.098	360 (86.3)	359 (86.1)	0.920	0.007
Non-valvular atrial fibrillation, n (%)	89 (20.7)	165 (8.4)	< 0.001	0.354	80 (19.2)	76 (18.2)	0.722	0.025
History of previous stroke, n (%)	144 (33.4)	453 (23.0)	< 0.001	0.232	138 (33.1)	130 (31.2)	0.553	0.041
History of congestive heart failure, n (%)	192 (44.6)	588 (29.9)	< 0.001	0.307	184 (44.1)	181 (43.4)	0.834	0.015
Revascularization due to Acute MI, n (%)	94 (21.8)	651 (33.1)	< 0.001	0.255	94 (22.5)	84 (20.1)	0.398	0.059
Revascularization after coronary CTA, n (%)	179 (41.5)	420 (21.3)	< 0.001	0.446	165 (39.6)	172 (41.3)	0.621	0.034
Duration after diagnosis of HCM, days	1142 (339–2224)	–	–	–	1144 (342–2223)	–	–	

Abbreviations: ASD, absolute standardized difference; CTA, computed tomography angiography; eGFR, estimated glomerular filtration rate; HCM, hypertrophic cardiomyopathy; MI, myocardial infarction; PS, propensity score.

### Comparison of clinical outcomes after revascularization

**Figure 2:**
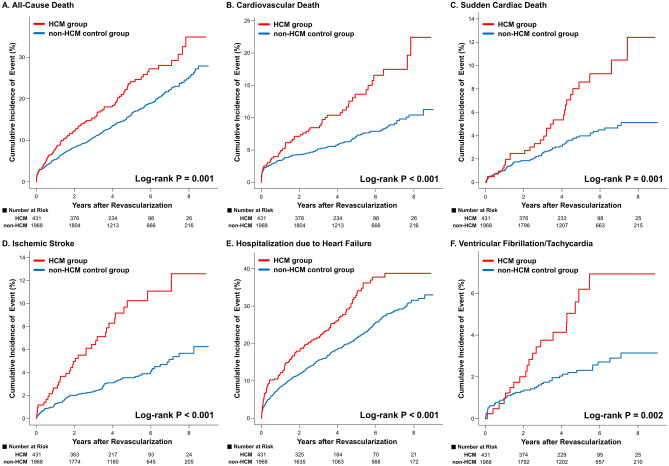
Cumulative incidence and risk of cardiovascular outcomes in HCM versus non-HCM Control Group. Kaplan–Meier curves of (**A**) All-cause death, (**B**) Cardiovascular death, (**C**) Sudden cardiac death, (**D**) Ischemic stroke, (**E**) Hospitalization due to HF, and (**F**) Ventricular fibrillation/tachycardia in the HCM group versus non-HCM control group are presented. Abbreviations: HCM, hypertrophic cardiomyopathy; HF, heart failure.

The risk of all-cause death (log-rank *P* = 0.001), cardiovascular death (log-rank *P* < 0.001), SCD (log-rank *P* = 0.001), ischemic stroke (log-rank *P* < 0.001), hospitalization due to HF (log-rank *P* < 0.001), and ventricular fibrillation/tachycardia (log-rank *P* = 0.002) was significantly higher in the HCM group than in the non-HCM control group (Fig. 2). The results were consistent after multivariable Cox regression or PSM to adjust for potential confounding factors (Table 2; PSM-HR [95% CI] for all-cause death = 1.78 [1.29–2.46], *P* = 0.001; for cardiovascular death = 3.15 [1.87–5.32], *P* < 0.001; for SCD = 2.20 [1.16–4.18], *P* = 0.016; for ischemic stroke = 4.83 [2.24–10.42], *P* < 0.001; for hospitalization due to HF = 1.40 [1.07–1.82], *P* = 0.014) except for ventricular fibrillation/tachycardia (adjusted HR [95% CI] = 1.74 [0.84–3.58], *P* = 0.135). Although the cumulative incidence of ventricular fibrillation/tachycardia was numerically more than 2 times higher in the HCM group than in the non-HCM control group, the difference did not reach statistical significance after adjustment for covariates or PSM. Cumulative incidence of MI (PSM-HR [95% CI] = 0.74 [0.35–1.55], *P* = 0.425) and repeat revascularization (PSM-HR [95% CI] = 0.79 [0.56–1.11], *P* = 0.179) was also not different between the two groups.

**Table 2:** Incidence and risk of clinical outcomes.

	HCM group		Non-HCM control group							
	Event/N	IR-per 1000 p-y	Event/N	IR-per 1000 p-y	Unadjusted HR (95% CI)	*P*	*Adjusted HR (95% CI)	*P*	PS-Matched HR (95% CI)	*P*
All-cause death	99/431	53.6	351/1968	36.6	1.45 (1.16–1.82)	0.001	1.63 (1.29–2.07)	< 0.001	1.78 (1.29–2.46)	0.001
Cardiovascular death	55/431	29.8	142/1968	14.8	1.95 (1.43–2.67)	< 0.001	2.27 (1.63–3.15)	< 0.001	3.15 (1.87–5.32)	< 0.001
Sudden cardiac death	29/431	15.8	71/1968	7.4	2.07 (1.34–3.18)	0.001	1.90 (1.20–3.01)	0.007	2.20 (1.16–4.18)	0.016
Ischemic stroke	36/431	20.3	72/1968	7.7	2.58 (1.73–3.86)	< 0.001	2.38 (1.55–3.64)	< 0.001	4.83 (2.24–10.42)	< 0.001
Hospitalization due to heart failure	122/431	78.7	434/1968	50.4	1.51 (1.23–1.84)	< 0.001	1.41 (1.14–1.74)	0.002	1.40 (1.07–1.82)	0.014
Ventricular fibrillation/tachycardia	20/431	11.0	44/1968	4.6	2.28 (1.34–3.87)	0.002	1.63 (0.93–2.87)	0.090	1.74 (0.84–3.58)	0.135
Myocardial infarction	12/431	6.6	95/1968	10.3	0.61 (0.33–1.11)	0.106	0.66 (0.35–1.24)	0.200	0.74 (0.35–1.55)	0.425
Repeat revascularization	60/431	35.5	307/1968	36.2	0.94 (0.71–1.24)	0.673	0.91 (0.68–1.22)	0.524	0.79 (0.56–1.11)	0.179

*Multivariable Cox regression model included age, sex, low income, revascularization due to acute MI, revascularization after coronary CT angiography, hypertension, diabetes mellitus, dyslipidemia, atrial fibrillation, previous stroke, and congestive heart failure.

Abbreviations: CI, confidence interval; CT, computed tomography; HCM, hypertrophic cardiomyopathy; HR, hazard ratio; IR, incidence rate; MI, myocardial infarction; PS, propensity score; p-y-person-years; VF, ventricular fibrillation; VT, ventricular tachycardia.

### Results of 1-year landmark analysis of clinical outcomes

**Figure 3:**
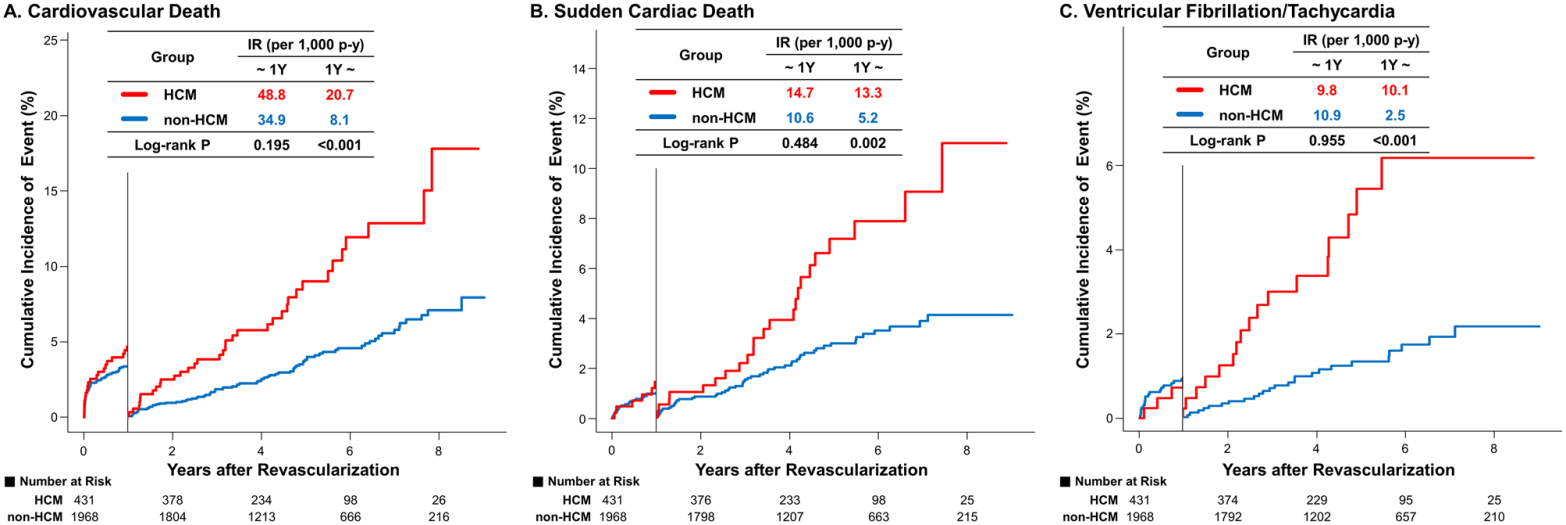
Landmark analysis of major cardiovascular outcomes in HCM versus non-HCM control group. Cumulative incidence and 1-year landmark analyses of (**A**) Cardiovascular death, (**B**) Sudden cardiac death, and (**C**) Ventricular fibrillation/tachycardia is presented. Abbreviations: HCM, hypertrophic cardiomyopathy; IR, incidence rate; p-y, person-year; Y, year.

For all-cause death (PSM-HR [95% CI] for 0–1-year = 2.10 [1.15–3.81], *P* = 0.015; for > 1-year = 1.67 [1.06–2.66], *P* = 0.029) and ischemic stroke (PSM-HR [95% CI] for 0–1-year = 5.14 [1.13–23.45], *P* = 0.035; for > 1-year = 4.47 [1.68–11.93], *P* = 0.003), the risks were significantly higher in the HCM group than in the non-HCM control group, regardless of the 1-year landmark analysis (Table 3). The risk of cardiovascular death (for 0–1-year, log-rank *P* = 0.195; for > 1-year, log-rank *P* < 0.001), SCD (for 0–1-year, log-rank *P* = 0.484; for > 1-year, log-rank *P* = 0.002), and ventricular fibrillation/tachycardia (for 0–1-year, log-rank *P* = 0.955; for > 1-year, log-rank *P* < 0.001) was significantly higher when the analysis was limited to the period after 1 year of index revascularization (Fig. 3). The risks of MI (PSM-HR [95% CI] for 0–1-year = 1.03 [0.36–2.93], *P* = 0.957; for > 1-year = 0.59 [0.20–1.77], *P* = 0.347) and repeat revascularization (PSM-HR [95% CI] for 0–1-year = 0.68 [0.38–1.19], *P* = 0.177; for > 1-year = 0.92 [0.59–1.44], *P* = 0.718) were the same in the HCM and the non-HCM control groups during the first year of revascularization as well as after 1 year of revascularization.

**Table 3:** Incidence and relative risk of clinical outcomes by 1-year landmark analysis.

	HCM group		Non-HCM control group							
	Event/N	IR-per 1000 p-y	Event/N	IR-per 1000 p-y	Unadjusted HR (95% CI)	*P*	*Adjusted HR (95% CI)	*P*	PS-Matched HR (95% CI)	*P*
All-cause death										
0–1-year	34/431	82.9	106/1968	56.0	1.48 (1.00–2.17)	0.048	1.76 (1.17–2.65)	0.007	2.10 (1.15–3.81)	0.015
1-year–	45/326	37.3	171/1552	26.1	1.47 (1.06–2.05)	0.022	1.65 (1.17–2.32)	0.004	1.67 (1.06–2.66)	0.029
Cardiovascular death										
0–1-year	20/431	48.8	66/1968	34.9	1.39 (0.84–2.30)	0.195	1.81 (1.06–3.08)	0.030	2.03 (0.95–4.33)	0.068
1-year–	25/326	20.7	53/1552	8.1	2.63 (1.63–4.24)	< 0.001	2.81 (1.71–4.61)	< 0.001	4.98 (2.03–12.22)	< 0.001
Sudden cardiac death										
0–1-year	6/431	14.7	20/1968	10.6	1.39 (0.56–3.45)	0.484	1.14 (0.44–3.00)	0.787	1.53 (0.43–5.43)	0.508
1-year–	16/326	13.3	34/1552	5.2	2.55 (1.40–4.62)	0.002	2.51 (1.34–4.71)	0.004	2.90 (1.12–7.47)	0.028
Ischemic stroke										
0–1-year	11/431	27.3	25/1968	13.3	2.04 (1.01–4.15)	0.048	1.73 (0.81–3.73)	0.160	5.14 (1.13–23.45)	0.035
1-year–	20/326	16.9	40/1552	6.2	2.70 (1.57–4.62)	< 0.001	2.53 (1.43–4.46)	0.001	4.47 (1.68–11.93)	0.003
Hospitalization due to heart failure										
0–1-year	50/431	132.6	161/1968	89.2	1.47 (1.07–2.01)	0.018	1.22 (0.87–1.71)	0.247	1.36 (0.89–2.09)	0.160
1-year–	67/326	62.2	244/1552	39.9	1.56 (1.19–2.05)	0.001	1.57 (1.18–2.08)	0.002	1.46 (1.02–2.08)	0.039
Ventricular arrhythmia (VT/VF)										
0–1-year	4/431	9.8	19/1968	10.1	0.97 (0.33–2.85)	0.955	0.52 (0.17–1.64)	0.266	0.61 (0.15–2.55)	0.498
1-year–	13 / 326	10.9	16/1552	2.5	4.31 (2.07–8.96)	< 0.001	3.79 (1.75–8.23)	< 0.001	3.63 (1.18–11.13)	0.024
Myocardial infarction										
0–1-year	7/431	17.2	46/1968	24.5	0.70 (0.32–1.56)	0.384	0.91 (0.40–2.10)	0.829	1.03 (0.36–2.93)	0.957
1-year–	5/326	4.2	44/1552	6.8	0.59 (0.23–1.48)	0.256	0.53 (0.20–1.39)	0.196	0.59 (0.20–1.77)	0.347
Repeat revascularization										
0–1-year	21/431	52.4	130/1968	70.9	0.74 (0.47–1.17)	0.199	0.79 (0.49–1.28)	0.338	0.68 (0.38–1.19)	0.177
1-year–	36/326	32.0	164/1552	27.0	1.15 (0.80–1.65)	0.449	1.06 (0.72–1.55)	0.773	0.92 (0.59–1.44)	0.718

*Multivariable Cox regression model included age, sex, low income, revascularization due to acute MI, revascularization after coronary CT angiography, hypertension, diabetes mellitus, dyslipidemia, atrial fibrillation, previous stroke, and congestive heart failure.

Abbreviations: CI, confidence interval; CT, computed tomography; HCM, hypertrophic cardiomyopathy; HR, hazard ratio; IR, incidence rate; MI, myocardial infarction; PS, propensity score; p-y-person-years; VF, ventricular fibrillation; VT, ventricular tachycardia.

### Subgroup analysis

**Figure 4:**
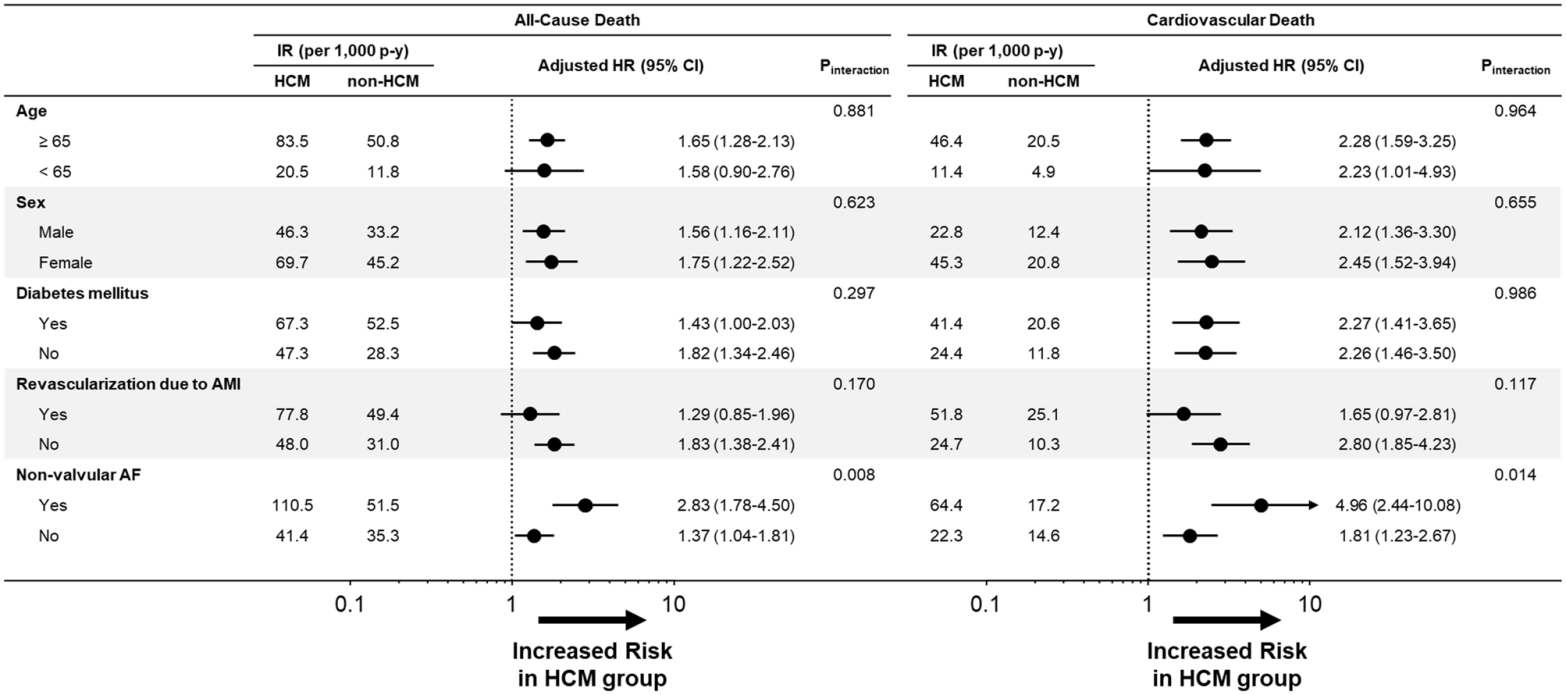
Subgroup analysis for all-cause death and cardiovascular death. Subgroup analyses across various exploratory subgroups for all-cause death and cardiovascular death are shown. Abbreviations: AMI, acute myocardial infarction; CI, confidence interval; HCM, hypertrophic cardiomyopathy; HR, hazard ratio; IR, incidence rate; p-y, person-year.

The risk of all-cause and cardiovascular death was further compared in the various subgroups (Fig. 4). A higher risk of mortality in the HCM group compared to the non-HCM control group was consistent across the subgroups by age (*P* for interaction = 0.881 for all-cause death and 0.964 for cardiovascular death), sex (*P* for interaction = 0.623 for all-cause death and 0.655 for cardiovascular death), the presence or absence of DM (*P* for interaction = 0.297 for all-cause death and 0.986 for cardiovascular death), or revascularization due to acute MI (*P* for interaction = 0.170 for all-cause death and 0.117 for cardiovascular death). However, the risk of both all-cause and cardiovascular death was prominently higher in the HCM group with coexistent non-valvular AF (*P* for interaction = 0.008 for all-cause death and 0.014 for cardiovascular death).

## Discussion

In this nationwide population-based study of 431 HCM patients and 1968 non-HCM matched controls who underwent coronary revascularization, we found that (1) the HCM group had a significantly higher risk of various cardiovascular events, including mortality, SCD, ischemic stroke, and hospitalization due to HF than the non-HCM control group, (2) the landmark analysis showed that the risk of cardiovascular death, SCD, and ventricular fibrillation/tachycardia was significantly higher in the HCM group compared to the non-HCM control group after 1 year of coronary revascularization, and (3) the co-existence of non-valvular AF significantly augmented the future risk of mortality, particularly from cardiovascular causes in the HCM group, compared to the non-HCM control group.

It was widely accepted that SCD, HF, and ischemic stroke are the leading causes of death in HCM^22^. Moreover, the co-morbidities that are unrelated to HCM have a greater impact on death from HF or stroke in HCM patients ≥ 60 years of age^23^. Concurrent ischemic heart disease, in particular, causes a significant deterioration in the natural course of HCM^2^. Patients with HCM frequently present with myocardial ischemia even in the absence of epicardial coronary artery stenosis, which may result from coronary microvascular dysfunction with decreased coronary flow reserve^1,6^. Hence, the presence of concomitant epicardial CAD makes HCM patients more vulnerable to additional ischemic burden^9^. However, previous studies have mostly focused on the prognosis of HCM patients with non-obstructive CAD. In the US national inpatient report of HCM patients with acute MI, 37% of patients with ST-segment elevation MI and 19% of patients with non-ST-segment elevation MI underwent coronary revascularization^24^. In a Taiwanese population-based cohort study, on the other hand, only 6.2% of patients with HCM and acute MI eventually underwent coronary stenting, and 82.5% were found to have no significant coronary artery stenosis^25^. However, no study has been published regarding the long-term cardiovascular outcomes in HCM patients with significant epicardial CAD who underwent coronary revascularization.

HCM accompanied by severe CAD was associated with a higher risk of death and SCD, with a mortality rate of 65.9, cardiac death rate of 43.4, and SCD rate of 21.0 per 1000 person-years (p-y), in a single-center report of 433 HCM patients who underwent coronary angiography^9^. However, this study was based on clinical practice during the 1970–1990s and did not reflect the contemporary management of CAD. For representative example, only 28.9% of patients with severe CAD underwent coronary revascularization. Although a head-to-head comparison of the two studies is not relevant given the different study periods and the different characteristics of the study population, the results of the present study (53.6 for all-cause death, 29.8 for cardiovascular death, and 15.8 for SCD per 1000 p-y, respectively) showed improved outcomes compared to those in the previous era. Additionally, when compared to the outcomes in all HCM patients (including those with and without CAD and coronary revascularization) in a study published previously (i.e., 9.7 for all-cause death, 5.8 for cardiovascular death, and 21.3 for hospitalization due to HF per 1000 p-y, respectively)^15^, the event rates of HCM patients with CAD and coronary revascularization were 3–5 times higher, even after managing epicardial CAD by coronary revascularization. Thus, CAD requiring revascularization in HCM patients is a key risk factor that considerably raises the risk of not only mortality, ischemic stroke, or hospitalization due to HF, but also SCD or ventricular fibrillation/tachycardia even in the contemporary era of HCM management.

Cardiovascular events after coronary revascularization usually occur within the first year^26^. The period beyond the first year of revascularization is referred to as the chronic phase and is associated with a substantial decrease in the incidence of adverse events when compared to the first year after coronary revascularization^26^. However, the current study found that the increased risk of HCM-related cardiovascular events (e.g., hospitalization due to HF, SCD, or ventricular fibrillation/tachycardia), but not of CAD-related events (e.g., MI or repeat revascularization), persisted long after the coronary revascularization in HCM patients with concomitant CAD, which may have increased the long-term mortality in this population.

We observed that the risk of both all-cause and cardiovascular death increased by nearly 3 and 5 times, respectively, in HCM patients with revascularized CAD and concomitant non-valvular AF compared to the non-HCM control group. HCM with concomitant AF is associated with serious adverse events such as HF and ischemic stroke, resulting in a worse prognosis^2,11,14^. In addition, irregular rhythm and frequent tachycardia in AF may aggravate the vulnerability to ischemia in HCM patients with CAD and may also result in a long-term detrimental effect on cardiac function^27^. A high burden of systemic atherosclerosis and cardio-metabolic risk factors in CAD patients may further augment the risk of ischemic stroke associated with AF^28,29^. In fact, the HCM patients of the current study presented with a relatively higher proportion of hypertension, DM, and dyslipidemia, when compared to those enrolled in the earlier study^30^. This was expected because we only enrolled HCM patients who underwent coronary revascularization. The inevitable use of antiplatelet medications following coronary revascularization may increase the risk of bleeding and subsequent mortality in AF patients who are already taking anticoagulants for stroke prevention^31^. Therefore, early detection of AF, prompt rate and/or rhythm control, appropriate combination of antiplatelet and anticoagulant, and aggressive risk factor modification may attenuate the future risk of adverse events in HCM patients with CAD. Further researches are warranted regarding this issue.

Given the detrimental effects of significant epicardial CAD in HCM patients, a thorough examination and screening for risk factors should be recommended at the time of HCM diagnosis and regularly thereafter. For risk factor management, physicians may follow the standards for secondary rather than primary prevention in patients with HCM considering their susceptibility to ischemic insult^1^. Owing to the limited diagnostic accuracy of exercise electrocardiogram or nuclear imaging in detecting significant CAD in patients with HCM, CCTA or invasive coronary angiography should be a primary diagnostic modality if a patient presents relevant symptoms or has a moderate to high pretest probability for CAD^1,2^.

In the present study, the proportion of patients with a history of congestive HF at the time of coronary revascularization was nearly half in the HCM group. This may imply a delay in the diagnosis of severe CAD in HCM patients, and therefore coronary revascularization may have been performed at a later stage of the disease or after the systolic function began to decline. Therefore, it may help improve the prognosis in HCM patients if adequate coronary revascularization is performed prior to the onset of subclinical cardiac dysfunction. Increased long-term risk of SCD or ventricular fibrillation/tachycardia beyond 1 year after coronary revascularization in HCM patients also underscores the importance of regular assessment of the need for implantable cardioverter-defibrillator implantation in this population at risk.

Several limitations should be acknowledged. First, the present study has an innate limitation that comes from its retrospective and observational nature. To mitigate this limitation, we performed multivariable Cox regression and PSM analysis. Nevertheless, residual or unmeasured confounding factors may affect the results. Second, since the working definition was used based on the ICD-10 diagnostic code from the claims data, misclassification bias and the possibility of under- or over-reporting cannot be completely excluded. Third, data on lifestyle risk factors such as smoking habits, was not available. Fourth, information on the location of hypertrophied myocardial segments or the existence of dynamic obstruction was not available in this database. Finally, because the results of the present study only reflect East Asian ancestry, our findings need to be replicated in other ethnic groups.

In conclusion, concomitant CAD requiring revascularization in HCM patients is a strong indicator of prognosis that synergistically increases the long-term risk of major cardiovascular events. Efforts should be taken for early detection of CAD, followed by prompt risk factor modification, and adopting updated management strategies for HCM. Further research should be undertaken to improve the prognosis of this specific HCM patients, the number of whom is progressively increasing in the contemporary era of HCM management.

## Supplementary Information

Supplementary Information.

## Data Availability

The data that support the findings of this study are available from the Korean National Health Insurance Service but restrictions apply to the availability of these data, which were used under license for the current study, and so are not publicly available. Data are however available from the corresponding author, Dr. Hyung-Kwan Kim, upon reasonable request and with permission of the Korean National Health Insurance Service.
