# Supplementary material for: Impact of coronary artery revascularization on long-term outcome in hypertrophic cardiomyopathy patients: a nationwide population-based cohort study
Source: Sci Rep. 2023 Apr 19;13:6412. doi: 10.1038/s41598-023-33344-3 (PMC10115788; doi:10.1038/s41598-023-33344-3)
Supplement: Supplementary file 1 — Supplementary Information. [file 41598_2023_33344_MOESM1_ESM.docx]

**Impact of Coronary Artery Revascularization on Long-term Outcome in Hypertrophic Cardiomyopathy Patients: A Nationwide Population-based Cohort Study**

Tae-Min Rhee, MD^a^, Hyung-Kwan Kim, MD, PhD^a^, Bong-Seong Kim, PhD^b^,
Kyung-Do Han, PhD^b^, Hyun-Jung Lee, MD^a^, In-Chang Hwang, MD^c^,
Heesun Lee, MD, PhD^d^, Jun-Bean Park, MD, PhD^a^, Yeonyee E. Yoon, MD, PhD^c^,
Yong-Jin Kim, MD, PhD^a^, Goo-Yeong Cho, MD, PhD^c^

^a^ Department of Internal Medicine, Seoul National University Hospital, Seoul, Republic of Korea

^b^ Department of Statistics and Actuarial Science, The Soongsil University, Seoul, Republic of Korea

^c^ Cardiovascular Center and Department of Internal Medicine, Seoul National University Bundang Hospital, Seongnam, Gyeonggi, Republic of Korea

^d^ Division of Cardiology, Department of Internal Medicine, Seoul National University Hospital Healthcare System Gangnam Center, Seoul, Republic of Korea

**SUPPLEMENTAL MATERIALS**

1. **Supplementary Table 1 (Table S1)**

**Table S1.** Working definitions of study population, comorbidities and outcomes

| **Variables** | **ICD-10 codes** | **Additional working definitions** |
| --- | --- | --- |
| *Definition for study population* |  |  |
| Hypertrophic cardiomyopathy | I42.1, I42.2 | Admission or outpatient clinic ≥ 1 AND  Registered in the Rare Intractable Diseases (RID) program (Code : V127) |
| Coronary artery bypass graft surgery | - | Admission ≥ 1 with claims for coronary artery bypass graft surgery (Procedure codes : O1640, O1641, O1647, O1648, O1649, OA640, OA641, OA647, OA648, OA649) |
| Percutaneous coronary intervention | - | Admission ≥ 1 with claims for PCI (Procedure codes : M6551, M6552, M6553, M6554, M6561, M6562, M6563, M6564, M6565, M6566, M6567, M6571, M6572, M6634, M6638) |
| Myocardial infarction | I21-I22 | Admission ≥ 1 with claims for invasive coronary angiography ≥ 1 (Procedure code : HA670, HA680, HA681, HA682) during admission |
| *Covariates^*^* |  |  |
| Diabetes mellitus | E11-E14 | 1) Admission ≥ 1 or outpatient clinic ≥ 2  AND  2) Minimum 1 prescription of anti-diabetic drugs (sulfonylureas, metformin, meglitinides, thiazolidinediones, dipeptidyl peptidase-4 inhibitors, α-glucosidase inhibitors, SGLT2-inhibitor, GLP-1 agonist, or insulin) |
| Hypertension | I10-I13, I15 | 1) Admission ≥ 1 or outpatient clinic ≥ 2 AND  2) Minimum 1 prescription of anti-hypertensive drug (thiazide, loop diuretics, aldosterone antagonist, alpha-/beta-blocker, calcium-channel blocker, angiotensin-converting enzyme inhibitor, angiotensin II receptor blocker) |
| Dyslipidemia | E78 | 1) Admission ≥ 1 or outpatient clinic ≥ 2 AND  2) Minimum 1 prescription of lipid-lowering medication (statin, ezetimibe, fenofibrate) |
| Non-valvular atrial fibrillation | I48 Exclusion: I05.0, I05.2, I05.9, Z95.2-Z95.4 | Admission ≥ 1 or outpatient clinic ≥ 2 |
| Stroke | I60-I69 | Admission ≥ 1 or outpatient clinic ≥ 2 |
| Heart failure | I50, I42.0, I11.0, I13.0, I13.2 | Admission ≥ 1 or outpatient clinic ≥ 2 |
| Acute MI | I21-I22 | Admission ≥ 1 with claims for invasive coronary angiography ≥ 1 (Procedure code : HA670, HA680, HA681, HA682) during admission |
| Coronary CT angiography | - | Admission ≥ 1 or outpatient clinic ≥ 1 with coronary CT angiography (Procedure code : HA474) |
| *Endpoints* |  |  |
| All-cause death | - | Obtained from the Korean governmental mortality database (Statistics Korea; KOSTAT, https://kostat.go.kr/portal/eng/index.action) |
| Cardiovascular death | I00-I99 | Obtained from the Korean governmental mortality database, using ICD-10 codes for the cause of death (Statistics Korea; KOSTAT, https://kostat.go.kr/portal/eng/index.action) |
| Sudden cardiac death | 1) I46, I49.0  OR  2) - | 1) Admission ≥ 1  OR  2) Admission ≥ 1 with claims for cardiopulmonary resuscitation (Procedure code : M1583, M1584, M1585, M1586, M1587, M5873, M5874, M5875, M5876, M5877) |
| Ischemic stroke | I63, I64 | Admission ≥ 1 with claims for brain CT or MRI (Procedure code : HA441, HA451, HA461, HA851 [Brain CT]; HE101, HE201, HE301, HE401, HE501 [Brain MRI]) |
| Hospitalization due to heart failure | I50, I11.0, I13.0, I13.2 | Admission ≥ 1 |
| Ventricular fibrillation/tachycardia | I47.0, I47.2, I49.0 | Admission ≥ 1 |
| Myocardial infarction | I21-I22 | Admission ≥ 1 with claims for invasive coronary angiography ≥ 1 (Procedure code : HA670, HA680, HA681, HA682) during admission |

*Covariates were defined by diagnoses during hospitalization or at outpatient clinic during the past 1 year.

Abbreviations: CT, computed tomography; ICD-10, International Classification of Diseases 10^th^ revision from the World Health Organization; MI, myocardial infarction; MRI, magnetic resonance imaging; RBC, red blood cell.
